# Lnc-EPB41-Protein Interactions Associated with Congenital Pouch Colon

**DOI:** 10.3390/biom8030095

**Published:** 2018-09-17

**Authors:** Sonal Gupta, Nidhi Gupta, Pradeep Tiwari, Saji Menon, Praveen Mathur, Shanker Lal Kothari, Sivaramaiah Nallapeta, Krishna Mohan Medicherla, Prashanth Suravajhala

**Affiliations:** 1 Department of Biotechnology and Bioinformatics, Birla Institute of Scientific Research (BISR), Statue Circle, Jaipur 302021, India; gupta.sonal1990@gmail.com (S.G); tiwari.pradeep30@gmail.com (P.T.); 2 Department of Biotechnology, Amity University Rajasthan, Kant Kalwar, Jaipur 303002, India; slkothari28@gmail.com; 3 Department of Biotechnology, IIS University, Mansarovar, Jaipur 302020, India; nidhinimesh@gmail.com; 4 Department of Chemistry, School of Basic Science, Manipal University, Jaipur 303007, India; 5 NanoTemper Technologies, World Trade Centre, Bengaluru 302017, India; menon.saji@gmail.com (S.M.); myresearchbin@gmail.com (S.N.); 6 Department of Paediatric Surgery, SMS Medical College, JLN Marg, Jaipur 302004, India; azadanitaashu@gmail.com

**Keywords:** long non coding RNA, whole exome sequencing, protein interaction, congenital pouch colon, microscale thermophoresis

## Abstract

Congenital Pouch Colon (CPC) is a rare anorectal anomaly common to northwestern India, specifically Rajasthan. Despite efforts to understand the clinical genetic makeup of CPC, no attempt on identifying non-coding RNAs was done. We have earlier reported CPC’s rare variants from whole exome sequencing (WES) across 18 affected samples in a total of 64 subjects. A Smith–Waterman algorithm was used to infer a couple of lncRNAs from WES samples of CPC with predictions from the Noncode database. Further screening and quantification using polymerase chain reaction (PCR), we ascertained interactions using Micro Scale Thermophoresis (MST). We report the role of *lnc*-EPB41-1-1 shown to be promiscuously interacting with KIF13A substantiating their role in regulation.

## 1. Introduction

Congenital pouch colon (CPC) is a rare anorectal anomaly in which a part or the entire colon gets dilated in the form of pouch and communicates with the fistula through a genitourinary tract [1]. Congenital pouch colon is reported exclusively from northwestern India with 5–18% CPC cases known for total anorectal malformations [2]. Since 2005, several efforts have been made to understand the clinical genetic makeup of CPC, but no attempt has been made to study the genes responsible for the disease. Recently, we have screened 64 subjects out of which 18 affected samples were analysed using whole exome sequencing (WES) [3]. Identifying mutations and variants in both coding and non-coding regions affecting such phenotypes are not only of valuable interest towards clinical applications but are also important for enormous prognostic value for therapy. In addition, with the non-coding RNAs (ncRNAs) playing a role in regulation [4], the genetic variation observed in these regions are quite prominent to study phenotypes of interest [5,6]. The lncRNAs play important and diverse functions in gene regulation and protein interactions in a wide range of diseases [7,8]. Recent studies on whole exome or transcriptome analyses in neuronal, immunomodulatory and carcinomas have identified several ncRNAs, particularly, a few long non-coding RNAs (lncRNAs) explored from WES [9,10,11,12]. Such identification of ncRNAs from WES could be attributed to the sequencing chemistry or impact of library targeted on intergenic regions, which needs a careful reassessment. With a genetic basis for many Mendelian traits/rare diseases not being clear, there are challenges widely seen towards understanding the emergence of mutations for various phenotypes, viz.penetrance [13], dominance, age-of-onset [14] and expressivity [15], complex genetic and environmental interaction studies, etc. [16,17].

As the lncRNAs play a role in regulation [18], there is an enormous scope for ascertaining lncRNA-protein interactions. In this study, we inferred a lncRNA, viz.lnc-EPB41-1-1promiscuously interacting with six protein-coding genes and established its interaction with KIF13A, a 202 kDa trafficking protein [19] and a *bona fide* gene causal to CPC [3]. Furthermore, this kinesin family of microtubule-based motor proteins is known to regulate various processes such as mannose-6-phosphate receptor (M6PR) transport besides mediating melanosome biogenesis and cytokinesis. While this interaction was predicted between the two biomolecules, we have made an attempt to interpret this lncRNA-protein interaction pair using microscale thermophoresis (MST) [20].

## 2. Results and Discussion

**Figure 1:**
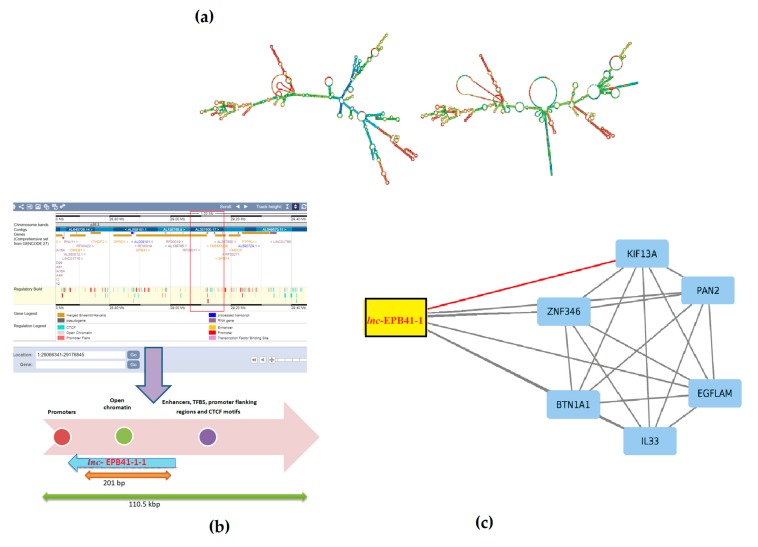
(**a**) RNAfold prediction showing base-pairing probabilities of the secondary structures of lncRNA wherein the centroid secondary structure (right)is shown to have a minimum free energy(MFE) of −322.60 kcal/mol indicating that the structure is in thermodynamic ensemble; (**b**) plausible role of lncRNA towards regulation with *lnc*-EPB41-1-1 known to be associated with open chromatin elements (OCE), promoters and enhancers; (**c**) *lnc*-EPB41-1-1 potentially shown to be interacting with KIF13A with the red edge indicating the predictions from lncPro and RNA Protein Interaction predictor (RPI-Pred).

To gain insight into the CPC genetics and interactions associated with the biomolecules, we have obtained a mean coverage of 100× with a sufficient depth of ca. 94% achieved for targeted exomes and intergenic boundaries from WES.A host of genes affecting the colon tissue besides CPC were found and the candidate genes and mutations were validated using Sanger sequencing from the 18 probands. The overall mutation density was checked with association of rare variants for CPC. We observed that the germline variants often tend to be false positives and are rare mutations and considered that a phenotype’s rarest events could not be achieved. From the WES, we identified *lnc*-EPB41-1-1 as a long intergenic non-coding RNA known to be interacting with KIF13A. The *lnc*-EPB41-1-1 is located in the intergenic regions of EPB41 (ENSG00000159023; chromosome 1:28880091-29459921) and transcribes a 1500nt ncRNA in the opposite orientation of EPB41. From the RNA fold predictions, we sought to determine that there are folds that induce changes in its secondary structure (Figure 1a).

## 3. Microscale Thermophoresis

**Figure 2:**
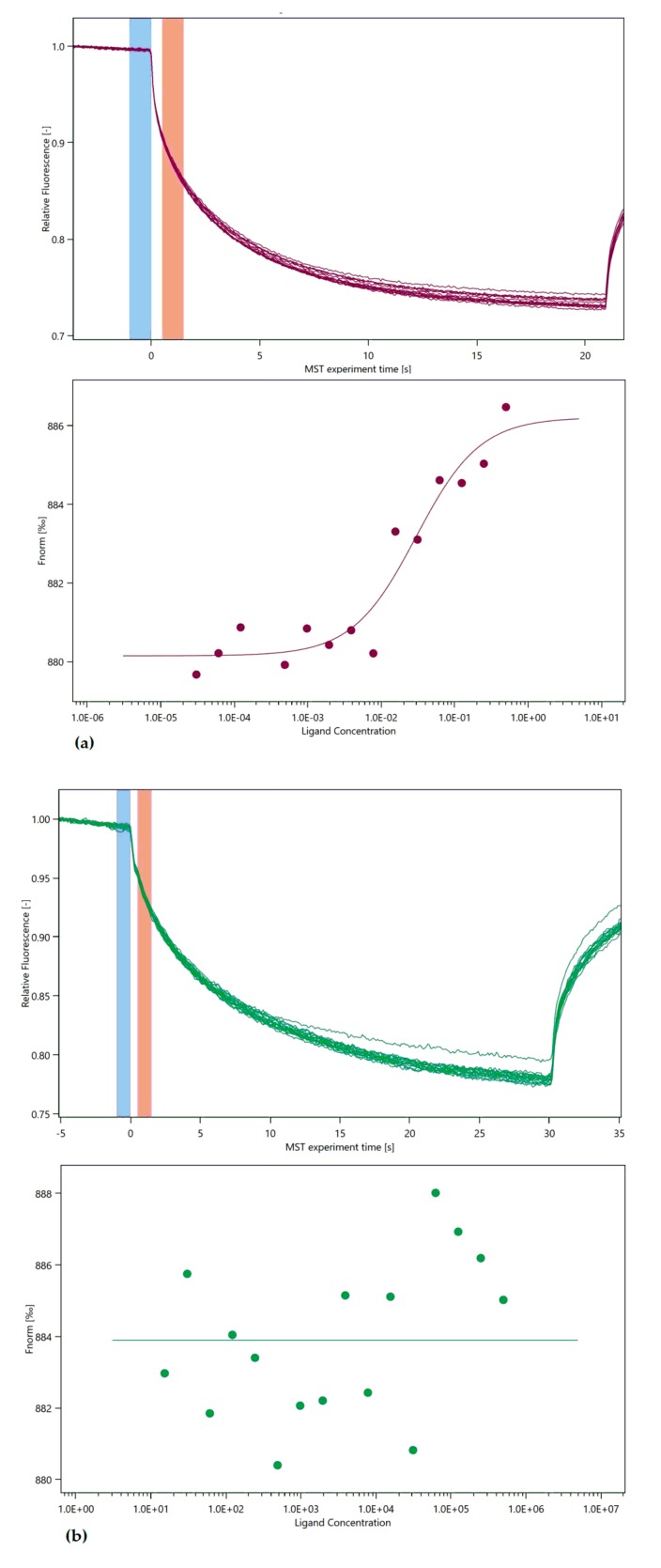
(**a**) the binding of fluorescently labelled protein to affected *lnc*-EPB41-1-1 is analysed with microscale thermophoresis (MST). RNA was titrated and change in the thermophoretic signal leads to binding affinity of 30 nM ± 11.6 nM with S/N: 10; (**b**) the interaction of labelled protein with wild type *lnc*-EPB41-1-1 is measured and MST showed no/weak binding under the tested conditions.

**Figure 3:**
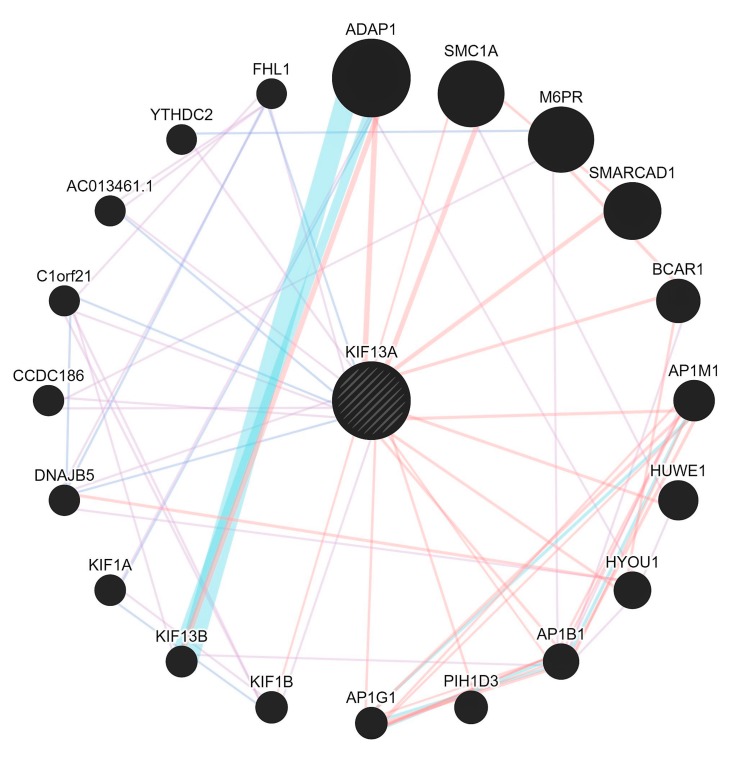
The pathway network map of KIF13A with pink edges (showing physical interactions), purple (co-expression) and violet (co-localization) and the blue thick edges showing the pathways.

When the unfolding profile of KIF13A protein was examined in the absence and presence of ligands, the binding of KIF13A to affected and wild type RNA was detected (Figure 2). Both interactions induced a shift of the unfolding transition to higher inflection temperatures (T_i_), indicating that an interaction has occurred. When the *lnc*-EPB41-1-1 with highest concentration (100 μM) was incubated with protein KIF13A (concentration kept constant), a binding was observed at the dissociation constant (Kd) value of 30 nM ± 11.6 nM, whereas wild type RNA interacted with KIF13A protein showed a weak/no binding. Once the binding of *lnc*-EPB41-1-1 with KIF13A was established, we assumed that there is a likely possibility of an interaction between these two biomolecules in affected tissue. While we demonstrated the interaction of *lnc*-EPB41-1-1 with KIF13A complementing bioinformatics predictions and MST, we argue that the bona fidelity of this interaction could be attributed to the following reasons:

1. The *lnc*-EPB41-1-1 harbors two potential open chromatin elements OCE, viz. ENSR00000003936 and ENSR00000003937 (E74-like factor 1, Ets family members respectively) linking it with a regulatory role [21]. In addition, the presence of transcription factor binding sites, promoters, CTCF motifs up/downstream provides evidence of its regulatory build for this region (Table S1) (Figure 1b). Furthermore, the primary role of CTCF motif is thought to be regulating the 3D structure of chromatin besides anchoring DNA to cellular structures, which influences the expression/repression of genes including *lnc*-EPB41-1-1. As *lnc*-EPB41-1-1 is one among a large number of conserved lncRNAs in mammalian/amniotic species [22], there is a growing significance that gene regulation could be associated with various phenotypes.
2. Evidence shows that *lnc*-EPB41-1-1 are known to be largely expressed in prostate and non-functional pituitary adenomas (NFPA) supporting its regulatory role in urological/colonic tissues as seen from a RNA-Seq expression profile [23]. In addition, when we checked the gene ontology pathways, it was observed that the KIF13A is involved in the manifestation of colon related disorders particularly the anorectal malfunction [24].
3. Due to the interactions in affected samples, there is a possibility that the pathways could be altered in CPC. We argue that with the mutations in essential genes tend to be causal for rare diseases [25] even as the mutations in non-coding genes could serve as drivers having higher prevalence rates. Furthermore, there appears to be selective pressure in those genes that share the pathways where they tend to be coexpressed, but not necessarily physically interacting/co-localizing (Figure 3).

## 4. Material and Methods

### 4.1. Identification of Long Non-Coding RNA

The University of Virginia (UVA) FASTA software (v36.6.8 version) [26] and NONCODE FASTA repository [23] were downloaded and the intergenic regions of the genes from WES samples were queried. Three lncRNAs were identified based on the query coverage e-value < 0 and the best possible hit in the form of NONHSAT002007 (Lncpedia accession *lnc*-EPB41-1-1) was obtained. The sequences were carefully checked for bidirectional blast hits and the lncRNA was visualized using an Ensembl genome browser for bona fidelity. The probable putative role of *lnc*-EPB41-1-1 was further checked by identifying the regulatory elements in the up/downstream regions of lncRNA (Figure 1c) followed by checking the prediction of interactions using lncPro [27] and RPI-Pred [28] for the six protein-coding genes.

### 4.2. Extraction of Biomolecules

**Figure 4:**
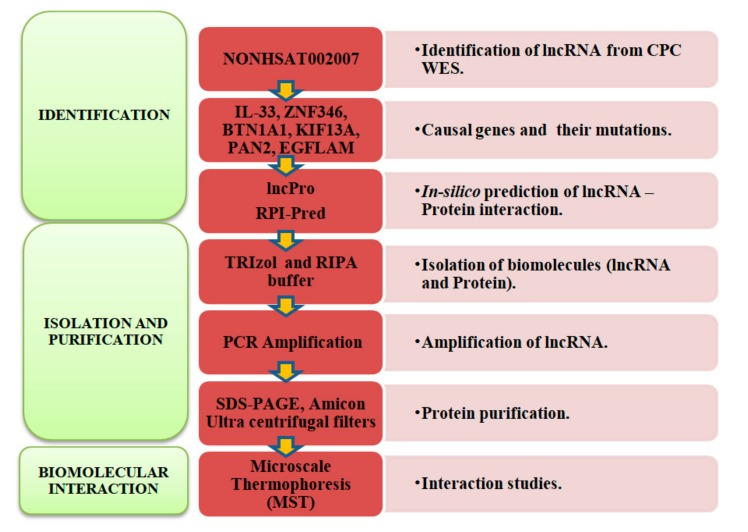
Flow chart demonstrating the methodology to characterize the lncRNA-protein interactions.

The samples were obtained after clearance from ethics committee of Sawai Man Singh Medical College and Hospital, Jaipur, India. Total RNA was isolated from 100 mg affected pouch colon and wild type (unaffected) colon tissues using TRIzol (Invitrogen™ 15596018, Carlsbad, CA, USA) according to the manufacturer’s protocol. RNA quality and quantity were checked through Biorad Experion^TM^ (Hercules, CA, USA) and a nanodrop spectrophotometer (Thermo Scientific, Waltham, MA, USA) respectively, with (A260/280) ratio above 1.9 considered as pure. Primers were synthesized for lncRNA (lnc-EPB41-1-1)-from Imperial Life Sciences (ILS), Gurugram, India (forward primer-5′AGAATCGCTTGAACCCAGGAGGC3′ and reverse primer- 5′ CAGATTGGGCTTAGACTCAGGAA 3′) and checked for primer dimerization by Gene runner software (6.5.48 × 64 beta version) [29]. PCR using *Pfu* high fidelity polymerase (Thermo Fisher Scientific, Waltham, MA, USA) was set with the following conditions: initial denaturation 99 °C (5 min), denaturation 94 °C (30 s), annealing 68 °C (30 s), extension at 72 °C for 45 s, cycles repeated for 40 cycles with final extension at 72 °C for 10 min. The amplification was checked on a 0.8% agarose gel and the desired PCR amplified product (1166 bp) was extracted using gel extraction kit (Qiagen, Hilden, Germany) for further downstream analysis. Total protein was isolated from affected and normal CPC samples using a RIPA buffer (5 M NaCl, 0.5 M ethylenediaminetetraacetate (EDTA) (pH 8.0), 1 M Tris (pH 8.0) and Triton X-100, 10% sodium deoxycholate, 10% sodium dodecyl sulfate SDS, 100 mM phenylmethylsulfonyl fluoride (PMSF)and protease inhibitor cocktail). Protein was quantified using a Bradford assay at 595 nm and ran on 10% SDS PAGE gel at 8 mA, 4 °C and overnight. A band size of approximately 202 kDa was excised from SDS PAGE and extracted through gel elution/renaturation buffer (20 mM Tris (pH 7.6), 1% Triton X-100, 1 mM EDTA, 2 mM dithiothreitol (DTT), 100 mM sodium chloride (NaCl) and incubated at 37 °C overnight while shaking. The band was further purified using Amicon Ultracel YM-100 by centrifuging at 10,000× *g* for 5 min at 4 °C and the purified filtrate was collected for further interaction analysis using MST (Figure 4).

### 4.3. Label Free Thermal Shift Analysis

The Tycho^TM^NT.6 system (a trademark of NanoTemper Technologies GmbH, Munich, Germany) offers a rapid and simple way to determine the protein quality and its interaction ability with other partners [30]. As we examined the protein-ligand interactions by performing a label-free thermal shift analysis, the Tycho usually heats up the sample from 35 °C to 95 °C in three minutes of time and determined the inflection point (T_i_) where protein unfolds. Further binding was detected by ligand induced changes in unfolded state.

### 4.4. Micro Scale Thermophoresis Affinity Measurements

Micro Scale Thermophoresis was performed to study biomolecular interaction using purified KIF13A protein. The purified protein was labelled using red fluorescent dye NT-647 according to the manufacturer’s instructions. Binding assays were performed with a Monolith NT.115 device (a trademark of NanoTemper Technologies GmbH, Munich, Germany) using standard treated capillaries. To improve the accuracy of the Kd determination, giving a fluorescence signal above 200 units, the concentration of labelled protein was kept to a minimum (100 nM). Equal amounts of labelled protein were titrated with varied ligand (RNA) concentration (2 µM to 0.06 nM). Furthermore, the change in the distribution of fluorescence upon heating was measured as a function of the concentration of the RNA-protein complex. Since migration of an individual molecule differs from migration of a molecule bound to ligand, the change in distribution of fluorescence was used to determine the ratio of free protein to protein bound to RNA. F_cold_ and F_hot_ were used to measure the fluorescence before and after heating, respectively. F_hot_/F_cold_ gave the normalized fluorescence with plots F_norm_ against the logarithmic concentrations of serially diluted ligand (RNA) giving sigmoidal binding curves.

## 5. Conclusions

We have demonstrated the application of MST for a rare CPC’s etiology from a WES sample by complementing *lnc*-EPB41-1-1’s bioinformatics predictions with MST. With this approach, the role of biologically relevant interactions that are otherwise regulatory could be shown not only for rare diseases such as CPC but any diseased phenotype of interest. We suggest that *lnc*-EPB41-1-1 bounded by regulatory elements might provide key evidence for causality of the disease. However, whether or not the lncRNAs targeting proteins are coexpressed is beyond the scope of this work, which can perhaps be considered for RNA-Seq studies in the future. Given the increasing numbers of lncRNAs recently reported in humans and the WES studies in rare diseases such as CPC, we may anticipate that more questions could be addressed in the future on the role of lncRNA-protein interaction pairs towards regulation.

## Supplementary Materials

The following are available online at http://www.mdpi.com/2218-273X/8/3/95/s1, Table S1: The *lnc*-EPB41-1-1 associated with regulatory elements, viz. transcription factor binding sites, promoters, CTCF motifs up/downstream. Supplementary Information: Thermal shift images of protein KIF13A with *lnc*-EPB41-1-1 using TychoTM NT.6.
